# Added Value of Rescue Devices in Intra-Arterial Thrombectomy: When Should We Apply Them?

**DOI:** 10.3389/fneur.2021.689606

**Published:** 2021-08-05

**Authors:** Cheng-Fu Ni, Sho-Jen Cheng, Cheng-Yu Chen, Tu-Hsueh Yeh, Kevin Li-Chun Hsieh

**Affiliations:** ^1^Department of Medical Imaging, Taipei Medical University Hospital, Taipei, Taiwan; ^2^Research Center of Translational Imaging, College of Medicine, Taipei Medical University, Taipei, Taiwan; ^3^Department of Radiology, School of Medicine, College of Medicine, Taipei Medical University, Taipei, Taiwan; ^4^Department of Neurology, Taipei Medical University Hospital, Taipei, Taiwan; ^5^Taipei Neuroscience Institute, Taipei Medical University, Taipei, Taiwan

**Keywords:** intra-arterial thrombectomy, stroke, rescue, thrombosuction, stent retreiver, large vessel occlusion

## Abstract

**Introduction:** Recent trials have demonstrated the superior efficacy of mechanical thrombectomy over other medical treatments for acute ischemic stroke; however, not every large vessel occlusion (LVO) can be recanalized using a single thrombectomy device. Rescue devices were proved to increase the reperfusion rate, but the efficacy is unclear.

**Objective:** In this retrospective study, we evaluated the efficacy of rescue therapy in different locations of LVO.

**Methods:** We analyzed the outcomes of mechanical thrombectomy from a prospective registry of consecutive 82 patients in Taipei Medical University Hospital. The reperfusion rate and the functional outcome were compared in patients who received first-line therapy only and patients who need rescue therapy.

**Results:** An 84.1% reperfusion rate was achieved in our cohort. We applied first-line stent retriever (SR) treatment in 6 patients, among which 4 (66.6%) achieved successful reperfusion. We applied a direct-aspiration first-pass technique (ADAPT) as the first-line treatment in 76 patients, among which 46 (60.5%) achieved successful reperfusion. Successful reperfusion could not be achieved in 30 cases (39.5%); therefore, we applied a second-line rescue SR for 28 patients, and reperfusion was established in 18 (64.3%) of them. These results revealed that the LVO in anterior circulation has a higher chance to respond to SR rescue therapy than posterior circulation lesions (68 vs. 33.3%, *P* < 0.001). Patients who received only first-line therapy exhibited significantly better functional outcomes than those who were also treated with rescue SR therapy (41.2 vs. 16.7%, *P* = 0.001). In addition, patients with LVO in the anterior circulation were found to have a higher probability of achieving functional independence than patients with posterior circulation lesions (10.7 vs. 0.0%, *P* < 0.001). The adjusted multivariate analysis revealed that successful reperfusion and treatment type (first-line or rescue therapy) were significantly related to a modified Rankin Scale (mRS) score at 90 days.

**Conclusion:** This study reveals that rescue SR therapy improves the reperfusion rate. Patients who require rescue SR therapy have a lower likelihood of functional independence. LVO in the anterior circulation responds better to rescue SR therapy and results in better functional outcomes than posterior circulation lesions.

## Introduction

Stroke is one of the leading causes of death and a primary source of disability among older adults worldwide. Ischemic stroke accounts for 80–85% of all strokes, with the anterior circulation stroke being the most frequent one. Intravenous alteplase (IV-tPA) administration is a first-line reperfusion therapy with proven efficacy (1); however, this therapy must be administered within 4.5 h of symptom onset. This short therapeutic time window is one of the most well-recognized limitations of this treatment. In addition, IV-tPA appears to be much less effective at recanalizing occlusions of the major intracranial arteries, but more than one-third of acute anterior circulation strokes are caused by major intracranial arterial occlusions (2). Recent trials and meta-analyses have demonstrated the greater efficacy of mechanical thrombectomy compared with other medical treatments for patients with acute ischemic stroke, with the balance of similar rates of adverse events (3–11). Stent retrievers (SRs), such as the Solitaire (Covidien, Plymouth, MN, USA) and Trevo (Stryker, Kalamazoo, MI, USA) retrievers, are predominantly employed in most trials. A direct-aspiration first-pass technique (ADAPT) is proposed to be a faster thrombectomy technique than the SR technique. Recent trials have demonstrated that ADAPT and the SR technique provide equivalent efficacy in terms of reperfusion rate and functional independence (12, 13); however, none of the aforementioned techniques can guarantee successful reperfusion. According to a meta-analysis comparing ADAPT with the SR technique, the reperfusion [obtaining a thrombolysis in cerebral infraction (TICI) score of 2b or 3] rate can be increased with second-line rescue devices (14); however, it is not clear whether the functional independence rate can also be increased by rescue therapy. In addition, whether the lesion location can affect the efficacy of rescue therapy remains unknown.

The present study investigated the efficacy of first-line and rescue mechanical thrombectomy therapies among patients with acute ischemic stroke. The relationships among the type of procedure, the time required for the procedure, the success of reperfusion, neurological outcome, and lesion location were analyzed.

## Materials and Methods

### Study Procedures

Data of the study cohort were retrospectively collected from a prospective registry of all consecutive patients who were referred for endovascular therapy to Taipei Medical University Hospital between August 2016 and December 2020. The institutional review board approved the use of the data. Patients with acute stroke treated with intra-arterial thrombectomy were recruited. Patients with incomplete clinical or radiographic data were excluded. Demographic variables, National Institutes of Health Stroke Scale (NIHSS) score at baseline, the timing of baseline, procedure time, and detailed procedural information were obtained from medical charts and intervention reports, filed by trained medical researchers and interventionists.

Criteria for thrombectomy eligibility comprised acute ischemic stroke from large vessel occlusion (LVO) within 16 h of symptom onset, an Alberta Stroke Program Early CT Score of >6, including if they had awakened from sleep with symptoms of a stroke. Perfusion imaging was performed for every patient whose time that they had last been well-known was longer than 6 h. Patients were eligible if they had an initial infarct volume (ischemic core) of <70 ml, a ratio of ischemic tissue to initial infarct volume of 1.8 or more, and an absolute volume of potentially reversible ischemia (penumbra) of 15 ml or more (15). The volume of the ischemic core and penumbral regions was estimated using CT perfusion scans and RAPID software (iSchemaView), an automated image post processing system. Penumbra size was estimated from the volume of the tissue to which the arrival of an administered contrast medium [time to a maximum of the residue function (Tmax)] exceeding 6 s (16).

All patients with an internal carotid artery or middle cerebral artery (M1, M2) occlusion and who fulfilled the inclusion and none of the exclusion criteria were eligible for intra-arterial thrombectomy. Patients with vertebral artery or basilar artery occlusion who presented symptoms within 16 h were also included without being required to meet the abovementioned perfusion scan criterion.

### Interventions

Patients with acute ischemic stroke were treated according to the American Heart Association/American Stroke Association guidelines for the early management of patients with acute ischemic stroke regarding endovascular treatment (15, 17). According to the guidelines, patients eligible for IV-tPA therapy were treated with it before undergoing the endovascular procedure. After LVO confirmation, patients were transported to an angiosuite equipped with a Siemens Artis zee biplane system (Siemens Healthcare, Erlangen, Germany). The procedure was performed under either local or general anesthesia. The selection of anesthesia type was left to the discretion of the attending interventional neuroradiologist and anesthesiologist. The brain vessels were accessed with a 6-F/90-cm guiding sheath (088 Neuron Max, Penumbra Inc., Alameda, CA, USA or Mach1, Boston Scientific, Marlborough, MA, USA). For the first-line ADAPT treatment, an aspiration catheter was navigated into direct contact with the thrombus. The Excelsior XT-27 microcatheter (Stryker Neurovascular, Fremont, CA, USA) over several types of 0.014 inch microwires and an ACE64 or ACE68 Penumbra aspiration system (Penumbra Inc., Alameda, CA, USA) with the original suction pump were employed for this technique. If the ADAPT technique failed to reach the occlusion site or achieve successful reperfusion (TICI 2b or 3) after at least three trials or passes, we applied an SR as the rescue therapy. For the rescue SR treatment, the microcatheter and the SR were navigated through the aspiration catheter used in the ADAPT technique. Continuous aspiration was performed while we retrieved the clots using manual aspiration through a 60 cc syringe attached to the 8Fr guiding catheter and penumbra aspiration tubing through the distal aspiration catheter. A Trevo XP ProVue (Stryker Neurovascular, Fremont, CA, USA) or a Solitaire SR (Medtronic, Dublin, Ireland) was employed in the first-line of rescue SR treatments. In cases of tight residual stenosis, a combination of procedures including extracranial stent implantation and angioplasty was used as necessary.

### Outcome Measures

A modified Rankin Scale (mRS) score for evaluating functional outcome at 90 days was assessed by certified neurologists. Favorable clinical outcome was defined as mRS score ≤ 2. Successful reperfusion was defined as a modified TICI score of 2b or 3 on digital subtraction angiography at the end of the procedure. All periprocedural and post procedural complications, including conversion from first-line devices to rescue therapy, were recorded in intervention and patient records. Symptomatic intracranial hemorrhage was defined as parenchymal hemorrhage at any site in the brain revealed by the CT scan, being compatible with documented neurological deterioration. Asymptomatic intracranial hemorrhage was defined as parenchymal hemorrhage at any brain site without deteriorated neurological function.

### Statistical Analysis

Descriptive statistics are expressed as means with SD or median with 25–75%. All data were tested with the D'Agostino-Pearson test to check whether samples were normally distributed or not. Non-parametric tests were applied for data that are not normally distributed. Multiple groups (first-line therapy only, successful and failure rescue SR therapy) were compared using the Kruskal-Wallis test and Dunn's multiple comparison test. Binary comparisons utilized chi-square or binomial tests for categorical variables, and the Mann-Whitney *U* test for continuous variables. Multivariate linear regression was performed to evaluate the relationship between mRS score at 90 days with predefined outcome prognosticators: successful reperfusion of not, lesion location (anterior or posterior LVO), and treatment type (first-line only or rescue therapy), adjusting for age, stroke severity (NIHSS) at baseline, symptom to reperfusion time, and IV-tPA therapy or not. Statistical analyses were performed using Prism (release 8.0, GraphPad Software Inc., La Jolla, CA, USA) and StatPlus: mac Pro (AnalystSoft Inc., Walnut, CA, USA). A *p* = 0.05 was considered statistically significant.

## Results

### Basic Demographics

Our cohort of 82 patients comprised 51 men and 31 women (Table 1). Their mean age was 72.1 ± 11.57 (33–93) years, and their initial median NIHSS score at presentation was 16 (12.0–20.0). In addition, 30.5% of them had atherosclerosis, and 6% of them had arterial dissection. The average time to puncture was 375.1 ± 241 min, and the overall reperfusion rate was 84.1%.

**Table 1:** Patient demographics with clinical and radiographic outcomes.

**Clinical Demographics and Disease Outcomes**			
**Data set**	**1st line therapy only (** ***N*** **=** **54)**	**Rescue SR therapy (** ***N*** **=** **28)**	***P*** **value**
Age	72.2 ± 10.8	71.9 ± 13.2	0.9
Gender (M:F)	34:20	17:11	0.7
Baseline NIHSS	15.8 ± 5.9	18.2 ± 7.8	0.2
Intravenous alteplase use	25.9% (17/54)	14.3% (4/28)	0.09
1st line techniques (SR:ADAPT)	6:48	0:28	
Atherosclerosis	25.9% (14/54)	38.3% (11/28)	0.5
Tortuous cervical carotid artery	24.1% (13/54)	28.6% (8/28)	0.7
Arterial dissection	1.8% (1/54)	14.3% (4/28)	0.03
Puncture to reperfusion time^*^	48.6 ± 33.2	78.0 ± 39.6	0.01
Symptom to reperfusion time^*^	334.2 ± 208.3	481.9 ± 290.4	0.02
**Clinical outcome**			
mRS median	3.0 (1.0–4.25)	4.5 (3.0–6.0)	<0.01
mRS 0–2 (%)	38.8% (21/54)	10.7% (3/28)	0.01
Reperfusion rate (TICI 2b &3)	60.5 or 94.4%^**^	64.3%	
**Complication**			
Mortality	13.1% (7/54)	21.4% (6/28)	0.32
sICH	14.8% (8/54)	10.7% (3/28)	0.6

*NIHSS, National Institutes of Health Stroke Scale; SR, stent-retriever; ADAPT, a direct aspiration first-pass technique: mRS, modified Rankin Scale; TICI, thrombolysis in cerebral infarction. sICH, symptomatic intracranial hemorrhage*.

* *Puncture to reperfusion time and event to reperfusion time do not include cases fail to be recanalized*.

** *Only include cases who did not receive further rescue therapy*.

### Detailed Thrombectomy Techniques

**Figure 1:**
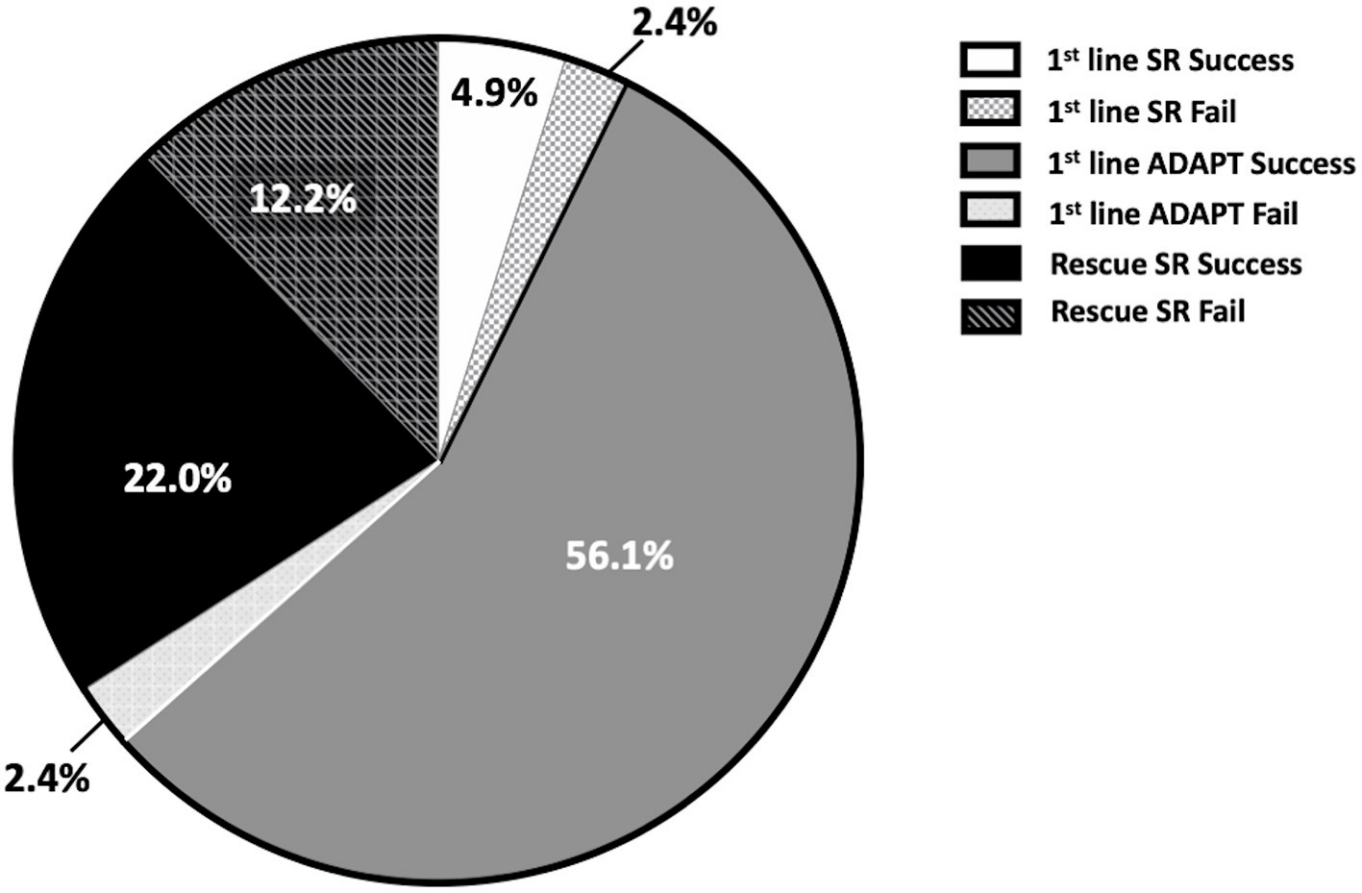
Distribution of successful and failed reperfusion in different techniques.

We applied first-line SR treatment in six cases, four of which (66.6%) resulted in successful reperfusion (Figure 1). We were unable to reach the occlusion site in two cases because of the tortuosity of the cervical carotid artery. We applied first-line ADAPT in 76 cases, 46 of which (60.5%) resulted in successful reperfusion. In 30 patients (39.5%), a TICI score of 2b or 3 was not achieved; therefore, we applied the second-line rescue SR treatment in 28, and reperfusion was achieved in 18 (64.3%) of these cases. We did not implement rescue therapy in the remaining two cases, because their vital signs were not stable and the time since symptom onset already exceeded 8 h after the failure of the first-line treatment. The overall reperfusion rate in the first-line ADAPT group and rescue SR group was 84.2% (53 of 76), which was higher than that in the first-line SR group. We have combined the first-line SR therapy and first-line ADAPT therapy into the first-line therapy only group for the following statistical calculation since there are only six cases in the first-line SR group.

### Functional Outcome at 90 Days

**Figure 2:**
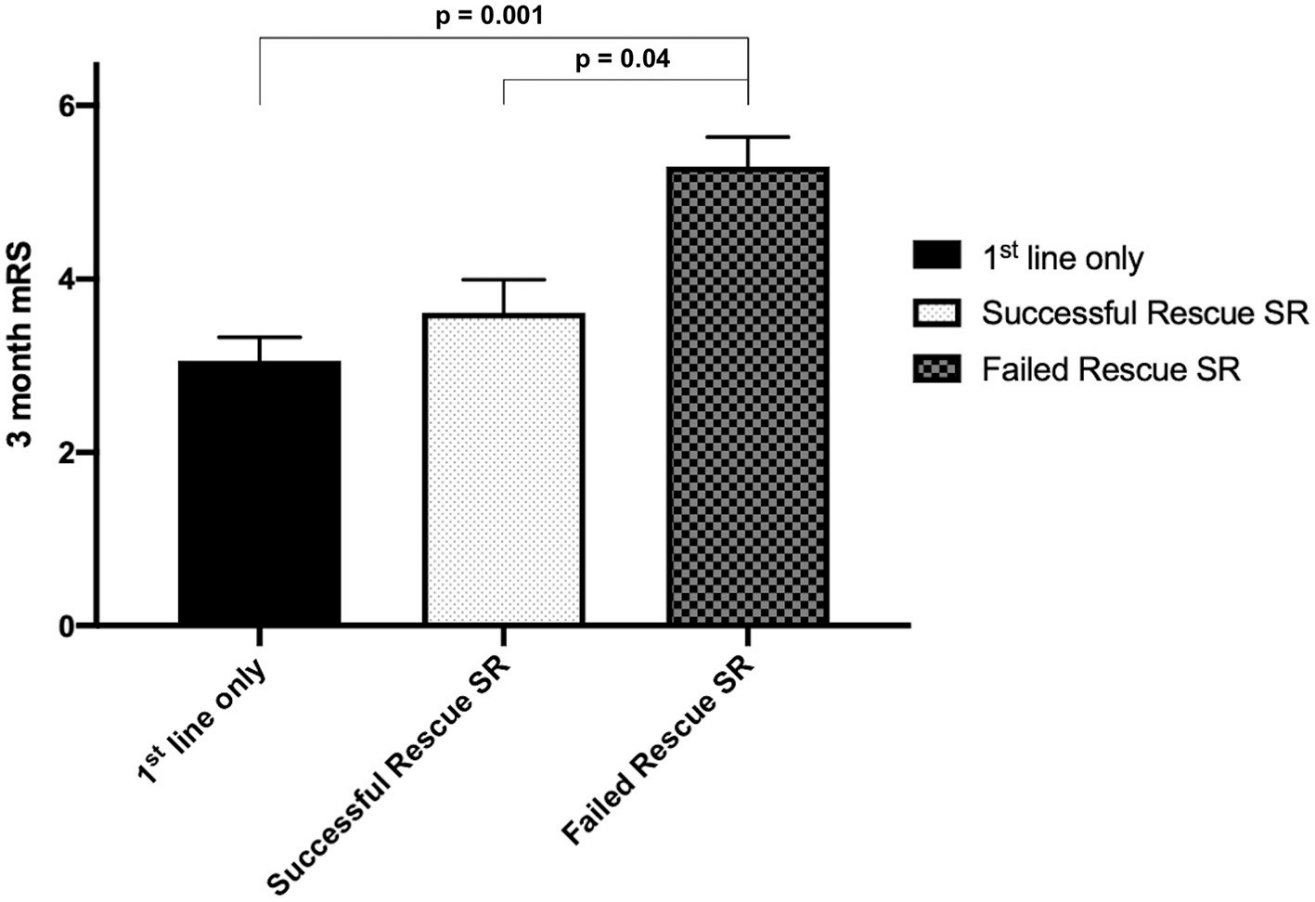
Mean Modified Rankin Scale (mRS) scores at 90 days in different groups. The mean mRS is higher in the failed rescue SR group. A significant difference is noted between patients receiving failed rescue SR therapy and those receiving first-line therapy only.

The median mRS score was 3.0 (1.0–4.25) in patients that received first-line therapy only, 3.0 (3.0–5.0) in patients that received successful rescue SR therapy, and 6.0 (4.75–6.0) in patients who failed rescue SR therapy. The mRS scores of patients with failed rescue SR therapy were significantly different from those of patients who received first-line only therapy or successful SR therapy (*P* = 0.001 and 0.04, respectively, Dunn's multiple comparison test; Figure 2). The overall functional independence rate is 29.2% in our cohort.

**Figure 3:**
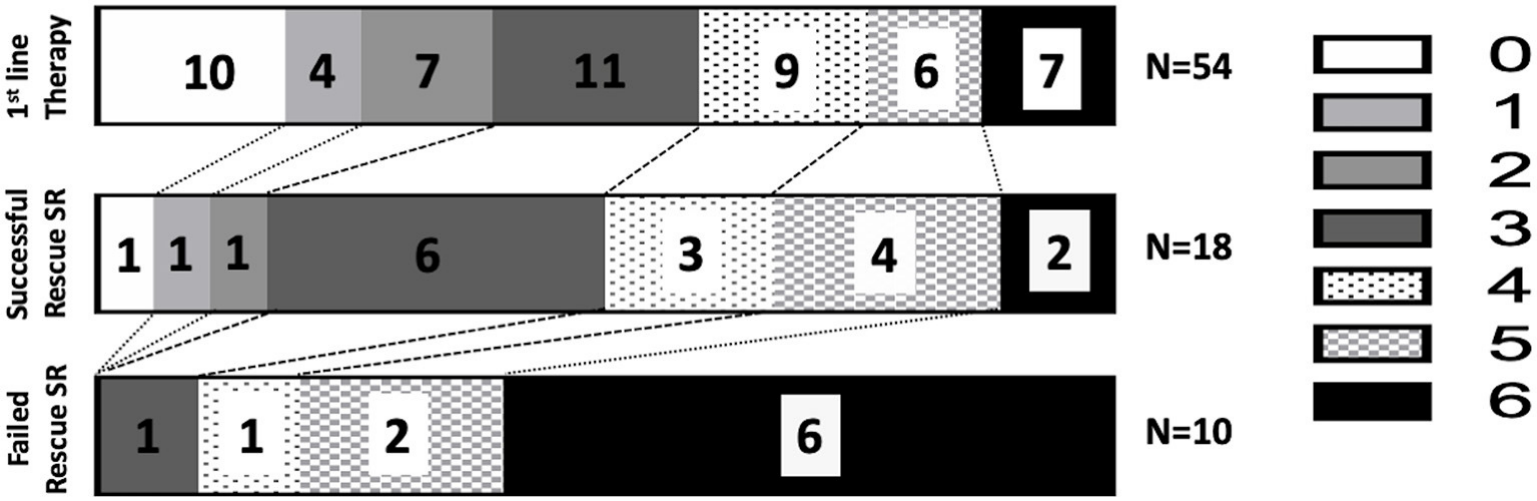
Modified Rankin Scale scores at 90 days in different groups. The functional independence rates (mRS score: 0–2) are 38.8% in the first-line ADAPT group, 16.7% in the successful rescue SR group, and 0% in the failed rescue SR group.

With regard to favorable clinical outcome rate in each group (mRS score of 0–2), 38.8% (21 out of 54) of patients receiving first-line therapy only and 10.7% (three out of 28) of patients in the rescue SR therapy group demonstrated functional independence, with an odds ratio of 5.3 (95% CI, 1.4–18.0, *P* = 0.001, Figure 3). The proportion of patients who achieved functional independence after successful reperfusion with first-line therapy only was 41.2%; this proportion was 16.7% for rescue SR therapy, with an odds ratio of 3.5 (95% CI, 0.93–12.33, *P* = 0.06).

### Procedure Time

The mean puncture to reperfusion time (puncture to reperfusion) for patients who received rescue SR therapy was 78.0 ± 39.6 min, which was longer than that for patients receiving first-line therapy only (48.6 ± 33.2 min, *P* = 0.01). The symptom to reperfusion time was significantly longer in the rescue SR group (481.9 ± 290.4 min) than in the first-line therapy only group (334.2 ± 208.3 min, *P* = 0.02).

We performed multivariate linear regression to evaluate the relationship between mRS score at 90 days with the predefined outcome prognosticators: successful reperfusion of not, lesion location (anterior or posterior LVO), and treatment type (first-line only or rescue therapy), adjusting for age, stroke severity (NIHSS) at baseline, symptom to reperfusion time, and IV-tPA therapy or not. The result revealed that only successful reperfusion (*p* = 0.03, odds ratio:1.16, 95% CI, 0.09–2.24) and treatment type (*p* = 0.04, OR: 0.97, 95% CI 0.02–1.94) were significantly related to mRS score at 90 days.

### Lesion Location in Rescue SR Therapy

For the 28 patients who received rescue SR therapy after failure of the first-line ADAPT therapy, the successful reperfusion rate was 64.3%. These results also revealed that LVO in anterior circulation (internal carotid artery and middle cerebral artery) has a higher chance to be recanalized by rescue SR therapy than posterior circulation lesion (68.0 vs. 33.3%, *p* < 0.001, Binomial test). It is also found that cases with anterior circulation lesions have a higher chance to have functional independence than cases with posterior circulation lesions (10.7 vs. 0.0%, *p* < 0.001) through rescue SR therapy.

### Complications and Mortality

The proportion of patients experiencing symptomatic intracranial hemorrhage was 10.7% (3 of 28) in the rescue SR therapy group, whereas 14.8% (8 of 54) in the first-line therapy group (*P* = 0.6). The mortality rate was 21.4% (6 of 28) in the rescue SR therapy group, whereas 13.1% (7 out of 40) in the first-line therapy only group (*P* = 0.32). No significant differences were found between the two groups.

## Discussion

In this retrospective observational study, we found a significantly higher likelihood of achieving a good functional outcome among patients who only received a first-line SR or ADAPT therapy than those who also received rescue SR therapy (38.8 vs. 10.7%). The adjusted multivariate analyses revealed that successful reperfusion and treatment type were significantly related to mRS score at 90 days. Patients with occlusion in anterior circulation have a better response to rescue SR therapy (68.0 vs. 33.3% reperfusion rate) and better functional outcome (10.7 vs. 0.0% functional independence rate) after rescue SR therapy compared with those with LVOs in the posterior circulation.

In previous reports (14, 18), ADAPT has been demonstrated to recanalize occlusions more quickly than the SR method. Because longer time in occlusion has been shown to put more quantities of tissue at the risk of becoming infarct core (19), we applied the ADAPT technique as first-line therapy in most cases. In this study, the mean procedure time and event to reperfusion time for patients who received rescue SR therapy were significantly longer than those receiving only first-line therapy, suggesting that patients required rescue SR therapy experience, longer tissue ischemia (512.3 min) than those received only first-line therapy (351.5 min). We hypothesize that prolonged tissue ischemia may be a cause of poor functional outcomes in cases requiring rescue therapy because a meta-analysis of recent clinical trials suggested that earlier reperfusion may result in better functional outcomes (8). However, variation still exists among individuals in the presence of native collaterals; the event to reperfusion time may not be the only indicator of the degree of tissue ischemia. It has been proposed that multiple passes of thrombectomy devices are associated with a higher risk for distal embolization and vessel injury (20). Several reports proved that first-pass reperfusion was associated with a more favorable clinical outcome than multiple passes, irrespective of different thrombectomy techniques (21–23). Therefore, more thrombectomy maneuvers may be one of the causes of worse outcomes in the rescue SR group. In addition, the rescue SR group has a higher proportion of intracranial atherosclerosis disease than the first-line therapy group (38.3 vs. 25.9%). It has been shown that intracranial stenosis is associated with more thrombectomy passes and worse disease outcomes (24, 25). Therefore, the necessity for rescue therapy may be an epiphenomenon, and better functional outcomes in patients who received first-line therapy only could result from multiple underlying causes, ranging from treatment effects to differing stroke pathologies. The application of IV-tPA is another important issue. More patients received IV-tPA treatment in the first-line therapy only group than those in the rescue therapy group (25.9 vs. 14.3%). Though previous reports have proved that alteplase treatment had no impact on the endovascular thrombectomy result (3–7, 26), it can be a confounding factor in this study. More research is required to further clarify these issues.

The functional independence rate in our cohort was not as high as the results in trials that applied the ADAPT technique (12, 13). The less favorable outcomes can be explained by three factors. First, we included patients who presented symptoms for longer than 6 h; such patients were not eligible for the ASTER and COMPASS trials. Although the inclusion criteria we applied were in accordance with those in the DIFFUSE 3 trial (11), many of the patients had larger infarct cores (>50 ml) than those in DIFFUSE 3. Second, we included patients with occlusion in the posterior circulation (vertebral or basilar arteries). Though many studies have demonstrated the efficacy of intra-arterial thrombectomy in the posterior circulation (27, 28), its benefit is still uncertain, even for patients with symptom onset within 6 h (15). We included 10 patients with posterior circulation LVO in our cohort. The successful reperfusion rate in this group was 80.0%; however, the functional independence rate at 90 days was only 20.0%. Third, a balloon guide catheter was not employed in this procedure. Regardless of treatment modality, it has been proved that the application of a balloon guide catheter in intra-arterial thrombectomy can improve the reperfusion rate and the first-pass success rate. In addition, it shortens the procedure time and leads to more favorable outcomes (29, 30). The major reason we did not use a balloon guide catheter is that the device is not reimbursed by the health insurance system in Taiwan.

Patients who received first-line SR or the ADAPT therapy only were a prognosticator detected in our cohort. Many other predictive factors of good functional outcome have been proposed in previous studies, including age, successful revascularization, parenchymal hemorrhage, baseline NIHSS score, anterior choroidal artery infarction, stroke subtype (intracranial atherosclerotic disease or embolism), posterior circulation Acute Stroke Prognosis Early CT Score, diffusion-weighted imaging lesion volume, glucose on admission, and hypersensitive C-reactive protein (31–35). The difference in reported factors may result from the heterogeneity of the study designs and patient demographics. More evidence and research are necessary to provide more accurate prognostic factors for intra-arterial thrombectomy outcomes.

Previous reports have shown that the devices required for aspiration techniques cost from US$4541 to US$5001 less than those for the SR technique (13, 14). ADAPT is considered to be a more cost-effective approach than SR while offering a similar reperfusion rate. When we applied rescue SR therapy after the first-line ADAPT treatment, the mean additional cost of different SRs was approximately US$6878, in the practice environment in the United States (13). Although the rescue SR therapy in this study provided a 64.3% successful reperfusion rate, only 10.7% of the patients recovered to be functionally independent after 3 months. These results also revealed that anterior circulation lesions had better functional outcomes after rescue SR therapy compared with posterior circulation lesions (10.0 vs. 0.0%). Because the choice of thrombectomy device and the decision to perform rescue therapy are both issues concerning value-based care (36), these results suggest uncertainty regarding whether applying rescue SR therapy for LVO in the posterior circulation is rational when the first-line ADAPT treatment cannot restore perfusion. More studies and evidence are warranted to address this issue.

## Limitations

This study has several limitations. First, this is a retrospective observational study with unbalanced patient numbers in the first-line SR and ADAPT groups. Second, SR therapy was the only procedure applied as rescue therapy; other treatments, such as intra-arterial recombinant tissue plasminogen activator, intra-arterial glycoprotein IIb/IIIa inhibitors, and intracranial stenting, were not used in our cohort. Third, we only performed local aspiration along with SR technique in rescue SR therapy; other techniques, such as CASPER (37) and SRLA (38), were not applied in our cohort. Finally, there were only 82 cases in this study, including 10 cases of LVO in the posterior circulation. Limited number in each subgroup is a constrain on the generalizability of result. Further study with a larger cohort is needed to validate this issue.

## Conclusion

This study revealed that patients with acute LVO stroke who are treated with only first-line SR or ADAPT therapy have significantly better functional outcomes than patients undergoing rescue SR therapy. Patients with occlusion in the anterior circulation have better responses to rescue SR therapy and better functional outcomes compared with patients with LVO in the posterior circulation. More research is required to prove the cost-effectiveness of rescue therapy in different subgroups of LVO patients.

## Data Availability Statement

The raw data supporting the conclusions of this article will be made available by the authors, without undue reservation.

## Ethics Statement

The studies involving human participants were reviewed and approved by TMU Joint Institutional Review Board No: N201804066. Written informed consent for participation was not required for this study in accordance with the national legislation and the institutional requirements.

## Author Contributions

C-FN, S-JC, and KH contributed to conception and design of the study. C-YC and KH organized the database. KH performed the statistical analysis. KH and T-HY wrote the first draft of the manuscript. All authors contributed to manuscript revision, read, and approved the submitted version.

## Conflict of Interest

The authors declare that the research was conducted in the absence of any commercial or financial relationships that could be construed as a potential conflict of interest.

## Publisher's Note

All claims expressed in this article are solely those of the authors and do not necessarily represent those of their affiliated organizations, or those of the publisher, the editors and the reviewers. Any product that may be evaluated in this article, or claim that may be made by its manufacturer, is not guaranteed or endorsed by the publisher.
